# Experience of Metronidazole Triple Therapy After Clarithromycin Triple Therapy Failure for *Helicobacter pylori* Eradication in Korea

**DOI:** 10.3390/jcm13247658

**Published:** 2024-12-16

**Authors:** Chang-Min Lee, Seong-Je Kim, Jung-Woo Choi, Hyun-Chin Cho, Ok-Jae Lee

**Affiliations:** 1 Department of Internal Medicine, Gyeongsang National University College of Medicine, Gyeongsang National University Hospital, Jinju 52727, Republic of Korea; cmleesam@gnuh.co.kr (C.-M.L.); sjhope77@naver.com (S.-J.K.);; 2 Institute of Medical Science, Gyeongsang National University, Jinju 52727, Republic of Korea

**Keywords:** *Helicobacter pylori*, eradication, metronidazole, triple therapy, bismuth, quadruple therapy

## Abstract

**Background/Objectives:** Bismuth quadruple therapy (BQT) is recommended as the best second-line regimen after failure of first-line clarithromycin triple therapy (CTT) for *Helicobacter pylori* eradication. However, there are some limitations to this approach, including the lack of an appropriate sequel regimen after failure of BQT and complicated administration. Metronidazole triple therapy (MTT) is simple to administer, but it is not widely recommended. This study was conducted to determine the efficacy of MTT as second-line regimen for *H. pylori* eradication after failure of CTT. **Methods**: We retrospectively reviewed the medical records of the Korean patients with *H. pylori* infection who underwent second-line treatment after failure of first-line CTT from October 2013 to October 2019. The efficacy of MTT and BQT for *H. pylori* eradication was compared. **Results**: The eradication rate in the BQT group tended to be higher than that in the MTT group; however, the difference was not statistically significant (208/233, 89.3% versus 244/284, 85.9%, *p* = 0.287). Among 40 patients with second-line MTT eradication failure, 21 received the third-line BQT, and 15 showed successful eradication (15/21, 71.4%). In the men 70 years or older, the eradication rate of MTT was lower than that of BQT without statistical significance (75.8% versus 94.1%, *p* = 0.141). **Conclusions**: These findings suggested that MTT could be a second-line treatment option, reserving BQT for *Helicobacter pylori* eradication after first line CTT failure, except in elderly men 70 years or older.

## 1. Introduction

*Helicobacter pylori* (*H. Pylori*) is a bacterium that colonizes the stomach [1]. Although many people infected with *H. pylori* remain asymptomatic, it can cause chronic gastritis, peptic ulcer disease, gastric mucosa-associated lymphoid tissue (MALT) lymphoma, and gastric cancer [2,3]. In 1994, International Agency for Research on Cancer (IARC) recognized it as a major cause of gastric cancer and classified it as a class 1 carcinogen [4]. Therefore, *H. pylori* eradication is important for the prevention of gastric cancer by improving atrophic gastritis and lowering the incidence of metachronous early gastric cancer [5,6,7]. In addition, eradication can induce complete remission of MALT lymphoma and prevent recurrence of peptic ulcers [8,9].

However, *H. pylori* is one of the bacteria that are difficult to eradicate, because *H. pylori* can form biofilms, which are complex communities of bacteria surrounded by a protective extracellular matrix. Biofilms can make it difficult for antibiotics to penetrate and kill the bacteria. In addition, *H. pylori* has the ability to survive in the gastric acidic environment. So, two or more antibiotics are recommended with high-dose proton pump inhibitors (PPIs). High-dose PPIs maximize antibiotic activity against *H. pylori* by suppressing gastric activity. Recently, potassium channel inhibitors have been developed and are being used for eradication treatment in some regions, including Japan, and additional research is required. Another reason why eradication for *H. pylori* is difficult is antibiotic resistance [9].

Clarithromycin-amoxicillin triple therapy (CTT) has been used as a classical first-line regimen to eradicate *H. pylori*. However, the eradication rate of CTT has been gradually declining, owing to the increasing resistance of this pathogen to clarithromycin [10]. After failure of first-line treatment for eradication, second-line regimens differ depending on the country and region. In Japan, patients who fail to achieve eradication with the first-line CTT regimen are treated with second-line metronidazole-containing triple therapy (MTT) [11]. According to the Maastricht VI consensus published in 2022, the algorithm for empirical *H. pylori* eradication is different depending on clarithromycin resistance. Bismuth-containing quadruple therapy (BQT) or CTT is recommended as a first-line regimen in areas of high (>15%) or unknown clarithromycin resistance, and BQT or fluoroquinolone-containing triple/quadruple therapy is recommended as a second-line regimen after failure of first-line CTT. In the area of high (>15%) or unknown clarithromycin resistance, however, BQT is the first option as a first-line regimen [12].

Although Korea has recently been regarded as an area with high clarithromycin resistance, the guidelines revised in 2020 still recommend 14-day clarithromycin triple therapy as the standard first-line eradication for *H. pylori* infection [13]. However, CTT for 7 days is recommended as the first-line regimen only if the clarithromycin resistance test by PCR is negative. Furthermore, sequential therapy (standard dose PPI, amoxicillin 1 g twice daily for 5 days followed by standard dose PPI, clarithromycin 500 mg, and metronidazole 500 mg twice daily for 5 days) or concomitant therapy (standard dose PPI, clarithromycin 500 mg, amoxicillin 1 g, and metronidazole 500 mg twice daily for 10 days) could also be considered as the first-line regimen. However, while the difference of eradication rate between a sequential regimen and a non-bismuth quadruple regimen is similar, there are concerns about side effects. In addition, there is another concern about potential second-line regimens when first-line BQT fails. Therefore, CTT is still recommended as the first-line regimen, and BQT is recommended as the first-line regimen in cases of clarithromycin resistance. When first-line CTT fails, 14-day BQT is recommended as a second-line regimen, and quinolone therapy is recommended as a third-line regimen [10,14,15,16].

Until recently, BQT has been considered the most reliable eradication therapy after first-line CTT fails. So, in Korea, BQT is generally chosen as the second-line regimen, despite some concerns. For example, BQT is associated with low safety and compliance. Specifically, the tetracycline contained in BQT is not commonly used for the treatment of infections other than *H. pylori,* owing to the induction of various complications [17,18]. Moreover, BQT is associated with low compliance because of the need for administration of multiple drugs with different dosing frequencies. Additionally, there is no proper sequel regimen after failure of BQT as a second-line treatment.

On the other hand, MTT is not recommended as the first-line regimen in Korea because the resistance to metronidazole is considered to be high in Korea. In addition, another worrisome reason is that exposure to metronidazole may reduce the effectiveness of metronidazole in subsequent BQT. However, reports on the eradication rate of MTT are rare in Korea, and it has not been confirmed whether the success rate of subsequent BQT decreases after the MTT failure for *H. pylori* infection.

In Korea, where the resistance to clarithromycin and metronidazole is high, there have been no studies on the eradication rate of MTT as a second-line regimen in patients with *H. pylori* infection. It is also unclear whether the use of metronidazole as a second-line treatment may affect the success rate of third-line BQT. Therefore, this study was conducted to evaluate the efficacy or usefulness of the MTT regimen in South Korea as a second-line treatment for *H. pylori* gastritis that failed first-line eradication therapy, in comparison to the BQT regimen.

## 2. Materials and Methods

### 2.1. Study Population

**Figure 1:**
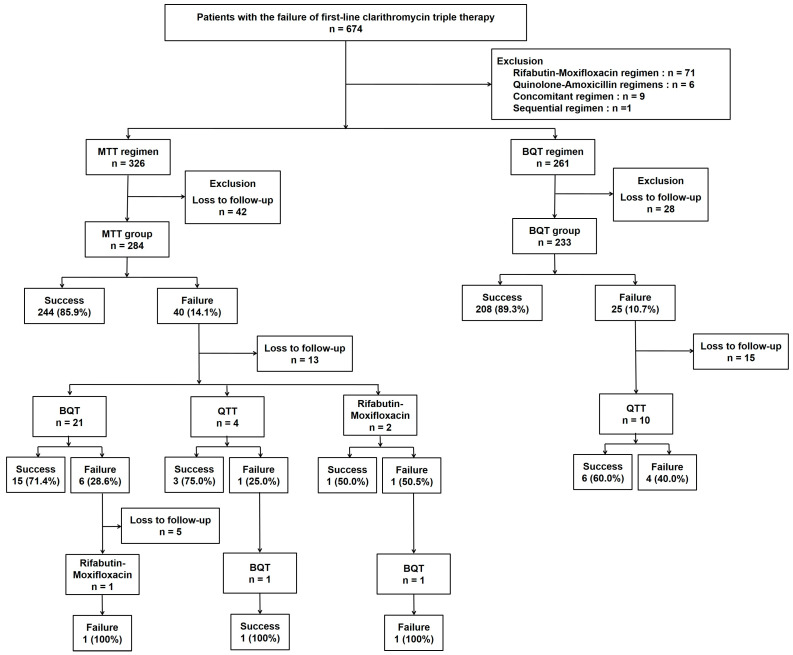
Flow chart of enrollment. Metronidazole triple therapy, MTT; bismuth quadruple therapy, BQT; quinolone triple therapy, QTT.

This study was a retrospective single center analysis conducted in Gyeongsang National University Hospital, a tertiary center in Korea, from October 2013 to October 2019. We collected a total of 674 adult patients who underwent second-line treatment for *H. pylori* infection after failure of first-line CTT during the study period. Among these, 587 patients who had taken MTT (326 patients) or BQT (261 patients) were finally included in the study for analysis, and we excluded 87 patients treated with rifabutin-moxifloxacin therapy (RMT, 71 patients), quinolone triple therapy (QTT, 6 patients), concomitant regimen (9 patients), or sequential regimen (1 patient) for second-line treatment (Figure 1). Therefore, intention-to-treat (ITT) analysis included all study subjects, a total of 587 patients (326 with MTT and 261 with BQT), and per-protocol (PP) analysis included 517 patients (284 with MTT and 233 with BQT), excluding 70 patients lost to follow-up (42 with MTT and 28 with BQT). In the subgroup analysis according to age and sex, 517 patients were analyzed. The eradication rate of third-line treatment and the final overall eradication rate were evaluated by PP analysis.

All patients were diagnosed as having *H. pylori* infection by endoscopic biopsy with or without Giemsa staining or rapid urease tests. The success of eradication was assessed using one or more follow-up endoscopic biopsies with or without Giemsa staining as well as rapid urease tests and urea breath tests. For Giemsa staining or rapid urease tests, tissues were obtained from the antrum and corpus of the stomach. Experts in pathology evaluated the presence of *H. pylori* infection using Giemsa stain. Rapid urease tests were conducted using the commercialized CLO kit (ASAN Easy Test *Helicobacter pylori*, Asan Pharmaceutical Co., Ltd., Hwaseong-si, Gyeonggi-do, Republic of Korea). To perform the urea breath test, the participant was instructed to fast for at least 4 h. The urea breath test was conducted following a 4 h fasting period. Expiratory air was collected using a commercially available sample container provided by Otsuka, Japan. Next, the participant ingested 100 mg of ^13^C tablets from the same manufacturer and exhaled into the sample container again 20 min later. The test to assess the success of eradication was performed at least 4 weeks after completion of the eradication treatment.

The MTT regimen was composed of metronidazole 500 mg b.i.d., amoxicillin 1 g b.i.d., and proton pump inhibitors (PPIs, rabeprazole 20 mg or esomeprazole 40 mg) b.i.d. for 14 days. The BQT regimen was composed of bismuth 300 mg q.i.d., tetracycline 500 mg q.i.d., metronidazole 500 mg t.i.d., and PPIs b.i.d. for 14 days. QTT regimen was composed of levofloxacin 750 mg q.d, amoxicillin 1 g b.i.d, and PPIs b.i.d for 14 days. RMT was composed of rifabutin 300 mg b.i.d, moxifloxacin 400 mg q.d, and PPIs b.i.d for 7 days.

This study was conducted in accordance with the Declaration of Helsinki. The Institutional Review Board of Gyeongsang National University Hospital reviewed and approved the study protocol (approval no. 2020-03-037). The review board waived the requirement for informed consent due to the retrospective study design.

### 2.2. Statistical Analysis

For two-group comparisons, Chi-square tests or Fisher’s exact tests (for situations with small frequencies) were used for categorical variables, whereas Student’s *t*-tests were used for continuous variables. For univariate analysis, bivariate logistic regression analysis was used. Results with P values less than 0.05 were considered statistically significant. All statistical analyses were performed using SPSS 21.0 (SPSS Inc., Chicago, IL, USA).

## 3. Results

### 3.1. Patient Characteristics

The mean age of patients in the MTT group tended to be 1.5 years higher than that in the BQT group; however, there was no statistically significant difference (Table 1). Although the proportion of men tended to be lower in the MTT group than in the BQT group, this difference was not statistically significant. None of the other variables were significantly different between the two groups.

### 3.2. Predictive Factors for Successful Eradication

In a univariate analysis using logistic regression, none of the factors, including age, sex, or history of smoking, had significant effects on the success of the second-line treatment (Table 2).

### 3.3. Comparison of Second-Line Eradication Rates Between the MTT and BQT Groups

The success rate of eradication of MTT group was 74.8% (244/326) in ITT analysis and 85.9% (244/284) in PP analysis. On the other hand, the success rate of eradication of the BQT group was 79.7% (208/261) in ITT analysis and 89.3% (208/233) in PP analysis (Figure 1). The success rate of second-line eradication in the MTT group was slightly lower than that in the BQT group; however, there was no statistically significant difference in either the ITT analysis or PP analysis (*p* = 0.169 in ITT analysis; *p* = 0.287 in PP analysis).

### 3.4. Sub-Analysis of Second-Line Eradication Rates According to Age and Sex

**Figure 2:**
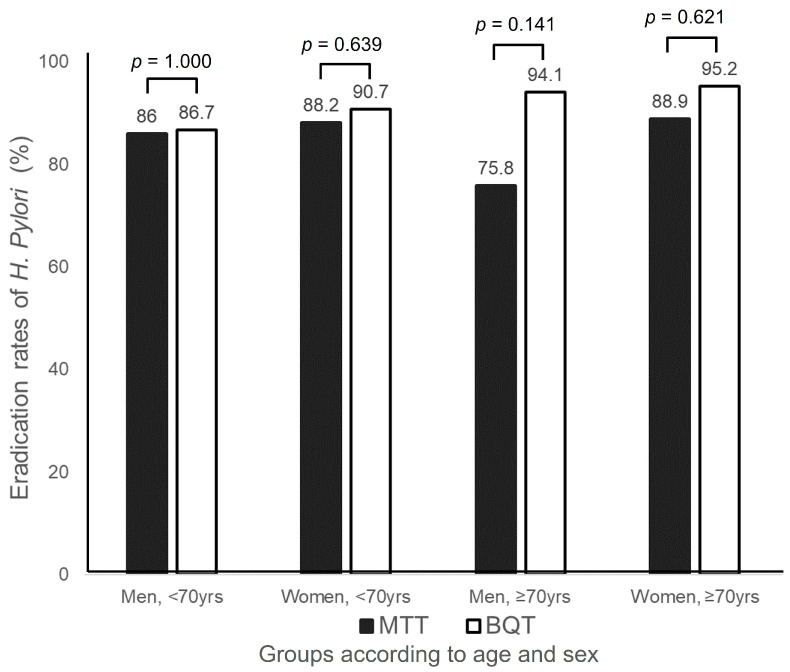
Comparison of second-line eradication rates for *Helicobacter pylori* between metronidazole triple therapy (MTT) and bismuth quadruple therapy (BQT) groups according to age and sex. MTT, metronidazole triple therapy; BQT, bismuth quadruple therapy.

For the 517 patients, the eradication rate was 89.7% in women, which was higher than that in men (85.6%). The eradication rates of BQT and MTT were 87.6% and 83.7%, respectively, in male patients, with a difference of 3.9% points, and were 91.7% and 88.3%, respectively, in female patients, with a difference of 3.4% points. In patients under 70 years old, the eradication rate of MTT was 87.1% (195/224), which was decreased by only 1.1% points from that (88.2%, 172/195) of the BQT (*p* = 0.768). In contrast, in elderly patients 70 years of age or older, the eradication rate of MTT was 13% points lower than in that of BQT (81.7%, 49/60 vs. 94.7%, 36/38, *p* = 0.074). In the subgroup analysis according to age and sex, the eradication rate of MTT was 18.3% points lower than that of BQT group in men 70 years of age or older; however, the difference was only 0.7% points between the eradication rates of two regimens in men under 70 years of age (Figure 2).

### 3.5. Eradication Rate of Third-Line Therapy After Failure of MTT

Out of the 40 patients who experienced MTT failure as a second-line regimen, 21 patients were prescribed BQT as a third-line regimen, and the infection was successfully eradicated in 71.4% (15/21) of cases (Figure 2). As for third-line eradication, QTT had an eradication rate of 75% (3/4), while RMT had an eradication rate of 50% (1/2).

### 3.6. Comparison of the Overall Eradication Rate According to the Second-Line Regimen

**Figure 3:**
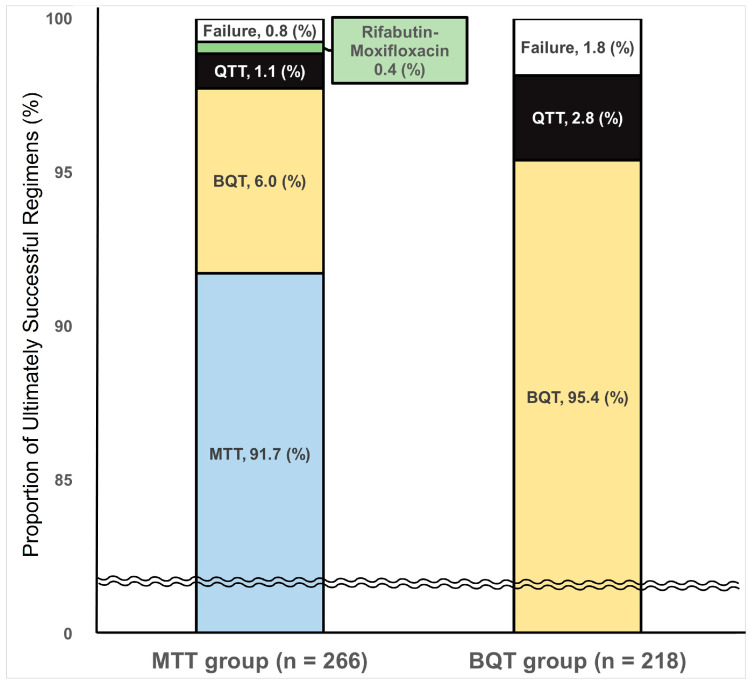
Proportion of the regimens that ultimately succeeded in eradication of *Helicobacter pylori* infection in the metronidazole triple therapy (MTT) and bismuth quadruple therapy (BQT) groups. MTT, metronidazole triple therapy; BQT, bismuth quadruple therapy; QTT, quinolone triple therapy (quinolone, amoxicillin, proton pump inhibitor). In the graph, the wavy midline break indicates omitted value for clarity.

Among the total of 486 patients who received up to the fourth-line rescue therapy regimens and were followed-up with, 480 showed successful eradication of *H. pylori,* and the overall eradication rate was 98.8%. The overall eradication rate was 99.2% (264/262) in the MTT group and 98.2% (214/218) in the BQT group (Figure 3). In the MTT group, the second-line MTT regimen successfully eradicated the infection in 85.9% (244/284) of patients, and 20 (7.6%, 20/262) out of the 40 patients with eradication failure after a second-line MTT regimen were finally eradicated with third- or fourth-line BQT regimens. Conversely, in the BQT group, the second-line BQT regimen successfully treated 89.3% (208/233) of patients; 6 (2.8%, 6/218) out of the 25 patients with eradication failure after a second-line BQT regimen had the infection eradicated with third-line QTT. Notably, three patients in the MTT group received a fourth-line regimen, resulting in a 33.3% (1/3) eradication rate, while no patients in the BQT group required a fourth-line regimen (Figure 1).

## 4. Discussion

In this study, the eradication rate of BQT as a second-line regimen for *H. pylori* infection was 79.7% in ITT analysis and 89.3% in PP analysis, which is close to the ideal value of 80% in ITT and 90% in PP analysis. Meanwhile, the eradication rate of MTT as a second-line regimen for *H. pylori* was 74.8% and 85.9% in ITT and PP analyses, respectively. BQT showed a higher eradication rate than MTT by 4.9% points in the ITT analysis and by 3.4% points in the PP analysis. However, these differences between the BQT and MTT did not reach statistical significance. Interestingly, the overall eradication rate investigated by counting the eradication rate up to the fourth-line regimen was 98.2% in the BQT group and 99.2% in the MTT group. Only 1.8% in the BQT group and only 0.8% in the MTT group ultimately failed to achieve eradication. Even if patients with the rifabutin-moxifloxacin regimen were excluded, the ultimate eradication rate of the MTT group was 98.8%, which is similar to that of the BQT group. This result shows the potential efficacy of MTT as a second-line regimen after failure of first-line CTT (Figure 3). Furthermore, 97.7% of the MTT group succeeded in *H. pylori* eradication with second-line MTT or third-line BQT, and this result implies that the second-line MTT does not decrease the ultimate eradication rate of a sequel regimen after failure of the second-line MTT.

Meanwhile, the lack of an appropriate third-line regimen after the second-line BQT failure may be another reason to consider BQT as the last regimen. Actually, in the present study, the success rate of the third-line quinolone triple therapy was only 60–75% as a rescue therapy after the second-line BQT failure. Based on our results, the MTT regimen could be considered as a second-line regimen instead of BQT, which could be reserved as the last rescue treatment option for patients for whom a BQT regimen is intolerable or difficult to take. In other words, the MTT regimen can be the bridge treatment option before the complicated BQT regimen is tried with these patients. BQT contains tetracycline, which is not used for any indication in recent clinical practice other than eradicating *H. pylori*. Tetracycline has serious adverse effects, including hepatotoxicity, whereas MTT is free from the risk of tetracycline toxicity [17,18]. Even if the eradication rate of MTT is not significantly different from that of BQT, it has fewer side effects and better compliance than the BQT regimen; MTT should be selected as a second-line regimen after failure of first line CTT. As MTT has strengths with regard to its easy usage, the same as with CTT, this regimen is expected to improve medication adherence, compared to BQT, which is a very complicated regimen to take.

In Korea, high resistance to metronidazole and clarithromycin has been reported [10,14,15,16]. In Western Gyeongnam province, in the southwestern region of Korea, where our center is located, the resistance rates to clarithromycin and metronidazole in children have been reported to be approximately 18.2% and 27.3%, respectively [19], although there are no data for adults. In regions with high resistance to clarithromycin and metronidazole, like Korea, the MTT regimen is typically considered to be ineffective for *H. pylori* eradication. In the preset study, however, the eradication rate of MTT was comparable to that of BQT, and this result supports that MTT can play a potential role as the second-line option after failure of first-line CTT. An MTT regimen was prescribed for 2 weeks, and its success rate was over 85% despite the high metronidazole resistance in Korea. This result may be attributed to the sufficient period of taking metronidazole. Actually, metronidazole resistance can be overcome by increasing the dose or expanding the period to 14 days [20,21]. Interestingly, an MTT regimen which does not include bismuth showed effects similar to those of BQT, although the dose of metronidazole was lower in the MTT than the BQT regimen (500 mg b.i.d. vs. 500 mg t.i.d.). This result suggests that the duration of metronidazole use may be a more important factor than the dose or concomitant use of bismuth.

Meanwhile, we did not find any factors influencing the *H. pylori* eradication rate in the univariate analysis; that is, the kind of second-line regimens did not influence *H. pylori* eradication (Table 2). When analyzing the data by age and sex, we could not find any significant difference in *H. pylori* eradication rates between the BQT and MTT groups in all populations, although there was a tendency towards higher eradication rates with BQT than MTT (Figure 2). Interestingly, the *H. pylori* eradication rate of MTT was lowest in elderly men 70 years of age or older; however, the eradication rates of the MTT and BQT groups were similar in men under 70 years old (*p* = 1.000, Figure 2). The difference in success rates between MTT and BQT was greater in men 70 years old and older than in male patients < 70 years of age (18.3% points vs. 0.7% points, Figure 2). Similarly, the difference in eradication rate between the MTT and BQT groups was larger in women 70 years and older than in female patients under 70 years (6.3% point vs. 2.5% point). However, the eradication rate of the MTT regimen in elderly women was comparable to that of BQT in female patients under 70 years. Taken together, these results imply that second-line MTT rather than BQT may be effective for eradication of *H. pylori* infection, except in men over 70 years of age. In other words, since the success rate of BQT was less than 90% in men under the age of 70, it is thought that MTT could be used as an alternative to BQT. Concerning the association between *H. pylori* infection, eradication, and age, it is suggested that *H. pylori* infection is more prevalent among the elderly population than in younger individuals, and that the efficacy of eradication treatment may be lower in older patients. As a result, stronger eradication treatments or additional testing may be necessary in older individuals [22,23].

One of the issues of MTT as a second-line regimen is the low eradication rate of the subsequent BQT after the failure of second-line MTT. As shown in this study, the eradication rate of third-line BQT was lower in patients who had already been exposed to metronidazole in second-line MTT than in those who had received second-line BQT (71.4% vs. 89.3%). However, the eradication rate of the other third-line QTT regimen was 50~75%, and it did not satisfy the requirement of an ideal regimen. Since any regimen in the third-line cannot guarantee an ideal eradication rate, a third-line BQT strategy might also be considered a strategy after the failure of second-line MTT. No matter how high the success rate of the second-line BQT itself, the eradication rate of the subsequent third-line QTT after the second-line BQT failure is only 60%, so the second-line BQT cannot be regarded as a perfect strategy. In addition, regarding the final overall eradication rate, the success rate of the MTT-BQT strategy was a little bit higher than that of the BQT-QTT strategy (97.7% vs. 95.4%).

The current study had some limitations. First, this was a retrospective study on patients treated for *H. pylori* infection before the recent guidelines were published. Therefore, there may have been a selection bias, owing to heterogeneity between the two groups. Indeed, the proportion of males tended to be higher in the BQT group than in the MTT group, although there was no statistical difference. However, the proportion of sex did not affect the second-line eradication rate in the univariate analysis, and the eradication rates of BQT and MTT showed a similar tendency in the stratified analysis by sex. Although the impact of this tendency on the results is expected to be very small, there may be a possibility of bias. Additionally, there were insufficient records of adverse effects in the chart review, and it was therefore difficult to compare the frequencies of complications between the two groups. However, the number of patients with loss of follow-up in the MTT group was not significantly different from that in the BQT group (12.9% (42/326) in the MTT group; 10.7% (28/261) in the BQT group; *p* = 0.423). In addition, the CYP2C19 genotype and susceptibility to *H. pylori* were not identified in each patient because such studies are not commonly performed in the clinical setting. This means that the possibility of bias due to the difference in metronidazole resistance rates between both groups cannot be ruled out. Another limitation of this study is the single-center nature of the study and the collection of data in one region of Korea. Therefore, this study is not representative of all regions in Korea. And the possibility of population changes due to the relatively long research period of 7 years can also cause bias. However, when analyzing the changes in eradication rates every 3 years in this study, there was no difference in the eradication rate by year in both groups. Finally, geographical factors should be considered. Antibiotic resistance varies geographically, and the resistance rates for the main antibiotics used for *H. pylori* eradication are quite different among the world’s continents [24]. It has been reported that the resistance rate to metronidazole is very high in certain regions, rendering its use impossible in those areas. The MTT strategy could not be used in some countries where metronidazole was not effective or suboptimal because of an extremely high resistance. Therefore, the MTT strategy cannot be universally applied worldwide.

Despite the high eradication rate of MTT shown in this study, these limitations do not provide sufficient evidence for the use of MTT as a second-line regimen in areas with high metronidazole resistance. However, these results are based on real-world clinical practice, where the sorts of antibiotics available for *H. pylori* eradication are very limited, raising fundamental questions about how to use discarded MTT. Therefore, large-scale randomized controlled trials are needed to verify the efficacy of MTT in the future. In addition, further research is needed to identify the conditions where MTT could be the most effective.

## 5. Conclusions

Our study suggests that MTT may be an attractive second-line regimen for the eradication of *H. pylori* after treatment failure with CTT. It also can make BQT a reserved last option, especially in male patients under 70 years, even in areas with high resistance to clarithromycin and metronidazole. Future large-scale randomized controlled trials are needed to verify MTT’s efficacy and identify optimal conditions for its use.

## Figures and Tables

**Table 1:** Baseline characteristics between metronidazole triple therapy (MTT) and bismuth quadruple therapy (BQT) groups.

Variables	MTT Group (n = 284)	BQT Group (n = 233)	*p* Value
Age, years	59.8 ± 12.3	58.3 ± 11.6	0.150
Sex, male	147 (51.8%)	137 (58.8%)	0.111
female	137 (48.2%)	96 (41.2%)	
Familial history of gastric cancer	25/207(12.1%)	11/135 (8.1%)	0.283
History of smoking	45/223 (20.2%)	38/172 (22.1%)	0.709
History of alcohol consumption	88/227 (38.8%)	64/167 (38.3%)	1.000
Kimura–Takemoto classification of chronic atrophic gastritis			0.022
Closed type	162/269 (60.2%)	108/218 (49.5%)	
Open type	107/269 (39.8%)	110/218 (50.5%)	
Intestinal metaplasia	81/269 (30.1%)	59/218 (27.1%)	0.446
Diagnosis			0.245
Peptic ulcer	155 (54.6%)	120 (51.5%)	
Gastritis	71 (25.0%)	49 (21.0%)	
Gastric neoplasm	54 (19.0%)	56 (24.0%)	
Hyperplastic polyp	3 (1.1%)	4 (1.7%)	
MALT * lymphoma	1 (0.4%)	4 (1.7%)	
Year of prescription			0.405
2013–2014	31 (10.9%)	20 (8.6%)	
2015–2016	55 (19.4%)	36 (15.5%)	
2017–2018	113 (39.8%)	107 (45.9%)	
2019	85 (29.9%)	70 (30.0%)	

* MALT lymphoma, mucosa-associated lymphoid tissue lymphoma.

**Table 2:** Logistic regression analysis of predictive factors for the successful eradication of *Helicobacter pylori* by the second-line regimen.

Variable	Odds Ratio (95% Confidence Interval)	*p* Value
Age (years)	1.00 (0.98–1.03)	0.774
Sex (male)	0.68 (0.40–1.16)	0.160
Familial history of gastric cancer	1.10 (0.41–2.97)	0.855
Smoking	0.69 (0.36–1.31)	0.254
Alcohol consumption	1.15 (0.64–2.08)	0.635
Kimura–Takemoto classification of chronic atrophic gastritis	0.99 (0.59–1.66)	0.969
Intestinal metaplasia	1.36 (0.75–2.46)	0.312
Regimen of second-line eradication (MTT ^1^ versus BQT ^2)^	0.73 (0.43–1.25)	0.254

^1^ MTT, metronidazole triple therapy; ^2^ BQT, bismuth quadruple therapy.

## Data Availability

The data presented in this study are available on request from the corresponding author. The data are not publicly available to protect patient privacy.

## Abbreviations

MALT, mucosa-associated lymphoid tissue; WHO, World Health Organization; IARC, International Agency for Research on Cancer; PPI, proton pump inhibitor; CTT, clarithromycin triple therapy; MTT, metronidazole triple therapy; BQT, bismuth quadruple therapy; RMT, rifabutin-moxifloxacin therapy; QTT, quinolone triple therapy; ITT, intention-to-treat; PP, per-protocol; PCR, polymerase chain reaction

## References

[1] Marshall B.J., Warren J.R. (1984). Unidentified curved bacilli in the stomach of patients with gastritis and peptic ulceration. Lancet.

[2] Graham D.Y., Dore M.P. (2016). Helicobacter pylori therapy: A paradigm shift. Expert Rev. Anti Infect. Ther..

[3] Hooi J.K.Y., Lai W.Y., Ng W.K., Suen M.M.Y., Underwood F.E., Tanyingoh D., Malfertheiner P., Graham D.Y., Wong V.W.S., Wu J.C.Y. (2017). Global prevalence of Helicobacter pylori infection: Systematic review and meta-analysis. Gastroenterology.

[4] International Agency for Research on Cancer (1994). Schistosomes, Liver Flukes and Helicobacter pylori.

[5] International Agency for Research on Cancer Helicobacter pylori Working Group (2014). Helicobacter pylori Eradication as a Strategy for Preventing Gastric Cancer.

[6] Choi I.J., Kook M.-C., Kim Y.-I., Cho S.-J., Lee J.Y., Kim C.G., Park B., Nam B.-H. (2018). Helicobacter pylori therapy for the prevention of metachronous gastric cancer. N. Engl. J. Med..

[7] Han S.J., Kim S.G., Lim J.H., Choi J.M., Oh S., Park J.Y., Kim J., Kim J.S., Jung H.C. (2018). Long-Term Effects of Helicobacter pylori Eradication on Metachronous Gastric Cancer Development. Gut Liver..

[8] Zullo A., Hassan C., Cristofari F., Andriani A., De Francesco V., Ierardi E., Tomao S., Stolte M., Morini S., Vaira D. (2010). Effects of Helicobacter pylori eradication on early-stage gastric mucosa-associated lymphoid tissue lymphoma. Clin. Gastroenterol. Hepatol..

[9] Graham D.Y., Lew G.M., Klein P.D., Evans D.G., Evans D.J., Saeed Z.A., Malaty H.M. (1992). Effect of treatment of Helicobacter pylori infection on the long-term recurrence of gastric or duodenal ulcer. Ann. Intern. Med..

[10] Lee J.Y., Kim N., Kim M.S., Choi Y.J., Lee J.W., Yoon H., Shin C.M., Park Y.S., Lee D.H., Jung H.C. (2014). Factors affecting first-line triple therapy of Helicobacter pylori including CYP2C19 genotype and antibiotic resistance. Dig. Dis. Sci..

[11] Asaka M., Kato M., Takahashi S., Fukuda Y., Sugiyama T., Ota H., Uemura N., Murakami K., Satoh K., Sugano K. (2010). Guidelines for the management of Helicobacter pylori infection in Japan: 2009 revised edition. Helicobacter.

[12] Malfertheiner P., Megraud F., Rokkas T., Gisbert J.P., Liou J.-M., Schulz C., Gasbarrini A., Hunt R.H., Leja M., O’Morain C. (2022). Management of Helicobacter pylori infection: The Maastricht VI/Florence Consensus Report. Gut.

[13] Jung H.-K., Kang S.J., Lee Y.C., Yang H.-J., Park S.-Y., Shin C.M., Kim S.E., Lim H.C., Kim J.-H., Nam S.Y. (2021). Evidence-based guidelines for the treatment of Helicobacter pylori infection in Korea 2020. Gut Liver.

[14] Kim J.J., Reddy R., Lee M., Kim J.G., El-Zaatari F.A.K., Osato M.S., Graham D.Y., Kwon D.H. (2001). Analysis of metronidazole, clarithromycin and tetracycline resistance of Helicobacter pylori isolates from Korea. J. Antimicrob. Chemother..

[15] Kim J.M., Kim J.S., Jung H.C., Kim N., Kim Y.-J., Song I.S. (2004). Distribution of antibiotic MICs for Helicobacter pylori strains over a 16-year period in patients from Seoul, South Korea. Antimicrob. Agents Chemother..

[16] Kim J.Y., Kim N., Kim S.J., Baik G.H., Kim G.H., Kim J.M., Nam R.H., Bin Kim H., Lee D.H., Jung H.C. (2011). Regional difference of antibiotic resistance of Helicobacter pylori strains in Korea. Korean J. Gastroenterol..

[17] Björnsson E., Lindberg J., Olsson R. (1997). Liver reactions to oral low-dose tetracyclines. Scand. J. Gastroenterol..

[18] Schultz J.C., Adamson J.S., Workman W.W., Norman T.D. (1963). Fatal liver disease after intravenous administration of tetracycline in high dosage. N. Engl. J. Med..

[19] Seo J.H., Jun J.S., Yeom J.S., Park J.S., Youn H.S., Ko G.H., Baik S.C., Lee W.K., Cho M.J., Rhee K.H. (2013). Changing pattern of antibiotic resistance of Helicobacter pylori in children during 20 years in Jinju, South Korea. Pediatr. Int..

[20] Graham D.Y., Hoffman J., el-Zimaity H.M., Graham D.P., Osato M. (1997). Twice a day quadruple therapy (bismuth subsalicylate, tetracycline, metronidazole plus lansoprazole) for treatment of Helicobacter pylori infection. Aliment. Pharmacol. Ther..

[21] Zhang X., Jiang A., Yu H., Xu J., Song Z., Sun L., Wang B., Ye L., Zhang L. (2016). Human lysozyme synergistically enhances bactericidal dynamics and lowers the resistant mutant prevention concentration for metronidazole to Helicobacter pylori by increasing cell permeability. Molecules.

[22] Pilotto A., Malfertheiner P. (2002). Review article: An approach to Helicobacter pylori infection in the elderly. Aliment. Pharmacol. Ther..

[23] Furuta T., Graham D.Y. (2010). Pharmacologic aspects of eradication therapy for Helicobacter pylori Infection. Gastroenterol. Clin. N. Am..

[24] Ierardi E., Giorgio F., Losurdo G., Di Leo A., Principi M. (2013). How antibiotic resistances could change Helicobacter pylori treatment: A matter of geography?. World J. Gastroenterol..

